# Genome-Wide DNA Methylation in Mixed Ancestry Individuals with Diabetes and Prediabetes from South Africa

**DOI:** 10.1155/2016/3172093

**Published:** 2016-07-31

**Authors:** Tandi E. Matsha, Carmen Pheiffer, Stephen E. Humphries, Junaid Gamieldien, Rajiv T. Erasmus, Andre P. Kengne

**Affiliations:** ^1^Department of Biomedical Sciences, Faculty of Health and Wellness Sciences, Cape Peninsula University of Technology, Cape Town 7535, South Africa; ^2^Biomedical Research and Innovation Platform, South African Medical Research Council, Cape Town 7505, South Africa; ^3^Centre for Cardiovascular Genetics, British Heart Foundation Laboratories, Institute of Cardiovascular Science, The Rayne Building, University College London, London WC1E 6JF, UK; ^4^South African National Bioinformatics Institute, University of the Western Cape, Cape Town 7535, South Africa; ^5^Division of Chemical Pathology and National Health Laboratory Service (NHLS), Faculty of Medicine and Health Sciences, University of Stellenbosch, Cape Town 7505, South Africa; ^6^Non-Communicable Diseases Research Unit, South African Medical Research Council, Cape Town 7505, South Africa; ^7^Department of Medicine, University of Cape Town, Cape Town 8000, South Africa

## Abstract

*Aims*. To conduct a genome-wide DNA methylation in individuals with type 2 diabetes, individuals with prediabetes, and control mixed ancestry individuals from South Africa.* Methods*. We used peripheral blood to perform genome-wide DNA methylation analysis in 3 individuals with screen detected diabetes, 3 individuals with prediabetes, and 3 individuals with normoglycaemia from the Bellville South Community, Cape Town, South Africa, who were age-, gender-, body mass index-, and duration of residency-matched. Methylated DNA immunoprecipitation (MeDIP) was performed by Arraystar Inc. (Rockville, MD, USA).* Results*. Hypermethylated DMRs were 1160 (81.97%) and 124 (43.20%), respectively, in individuals with diabetes and prediabetes when both were compared to subjects with normoglycaemia. Our data shows that genes related to the immune system, signal transduction, glucose transport, and pancreas development have altered DNA methylation in subjects with prediabetes and diabetes. Pathway analysis based on the functional analysis mapping of genes to KEGG pathways suggested that the linoleic acid metabolism and arachidonic acid metabolism pathways are hypomethylated in prediabetes and diabetes.* Conclusions*. Our study suggests that epigenetic changes are likely to be an early process that occurs before the onset of overt diabetes. Detailed analysis of DMRs that shows gradual methylation differences from control versus prediabetes to prediabetes versus diabetes in a larger sample size is required to confirm these findings.

## 1. Introduction

Deoxyribonucleic acid (DNA) methylation is a biochemical process catalyzed by DNA methyltransferase enzymes to covalently add a methyl group at the 5′ position of cytosine DNA nucleotides, creating 5-methylcytosine in CpG dinucleotides concentrated in gene promoters [1]. The CpG dinucleotides in gene promoters are not generally methylated; thus methylation at these sites is associated with changes in gene expression. Hypermethylation represses transcription, thereby reducing gene expression, while hypomethylation is associated with transcriptional activation of the affected genes [2, 3]. DNA methylation is the most characterized of the epigenetic processes, which also include histone modification, chromatin remodeling, and noncoding RNAs. Epigenetics, defined as mechanisms that affect gene transcription and/or expression in the absence of alterations to the DNA sequence, provides a plausible link between genetic and environmental determinants of health status. Current studies have shown congruence of epigenetic loci and gene polymorphisms in diseases [4–7]. In type 2 diabetes, for example, 19 single nucleotide polymorphisms (SNPs) associated with the disease introduced or removed potential sites for DNA methylation [7]. Furthermore, the genetic-epigenetic-environment link has been demonstrated in a report that showed an association between the nuclear-encoded gene, NADH dehydrogenase [ubiquinone] 1 beta subcomplex subunit 6 (NDUFB6) polymorphism, DNA methylation, age, and the expression of NDUFB6 in human skeletal muscle [4].

Estimates from the International Diabetes Federation (IDF) suggest that the population of people with diabetes is growing relatively faster in Africa compared to anywhere else [8]. Emerging evidence suggests that this process is not entirely accounted for by the traditional drivers of the diabetes epidemic. In mixed ancestry South Africans, for instance, who are largely overweight or obese and at high risk of diabetes [9], the distribution of traditional risk factors for diabetes is not appreciably different between individuals with diabetes and those without diabetes, while the accelerated deterioration of glucose tolerance status over time is not explained by the known powerful determinants of diabetes occurrence [10]. In this context, the contribution of emerging risk factors for diabetes including epigenetic changes has been postulated [11] but remains largely uninvestigated. It, therefore, became our primary aim to investigate nontraditional context specific diabetes risk factors and pathophysiological pathways underlying the excess risk of diabetes in this population. Herein, we report the South African mixed ancestry population's specific differentially methylated sites and metabolic pathways affected by DNA methylation in this population.

## 2. Subjects

Participants were members of a cohort study conducted in Ward 009, Cape Town, South Africa. The city of Cape Town defines Ward 009 as a mixed ancestry township formed in the late 1950s. According to the 2011 population census, its population stands at approximately 29,301 with an average household size of 4.84 individuals. The population is predominantly of mixed ancestry or coloured (76%) followed by black Africans (18.5%) and Caucasian and Asians who make only 1.5%. Most of the residents in this community have lived there for more than five years, while others have been there for their entire lives. The socioeconomic condition of the people is average with 37% of households having a monthly income of ZAR3, 200 or less. The recruitment of the Ward 009 cohort was initiated in April 2014, from which 3 individuals with screen detected diabetes, 3 individuals with prediabetes, and 3 individuals with normoglycaemia who were age-, gender-, body mass index- (BMI-), duration of residency-matched were selected for the current study. All participants were females.

## 3. Materials and Methods

### 3.1. Ethical Approval of the Study

This investigation is based on the Bellville South (Ward 009) cohort from Cape Town that has been approved by the Research Ethics Committees of the Cape Peninsula University of Technology and Stellenbosch University (resp., NHREC: REC-230 408-014 and N14/01/003). The study was conducted according to the Code of Ethics of the World Medical Association (Declaration of Helsinki). All participants signed written informed consent after all the procedures had been fully explained in the language of their choice.

### 3.2. Study Procedures

All participants received a standardized interview, blood pressure, and anthropometric measurements. Participants with no history of doctor-diagnosed diabetes mellitus underwent a 75 g oral glucose tolerance test (OGTT) as recommended by WHO [12]. Further, the following biochemical parameters were analyzed at an ISO 15189 accredited pathology practice (PathCare, Reference Laboratory, Cape Town, South Africa): plasma glucose, serum insulin, serum creatinine, total cholesterol (TC), high density lipoprotein cholesterol (HDL-c), triglycerides (TG), low density lipoprotein cholesterol (LDL), C-reactive protein (CRP), *γ*-glutamyl transferase (GGT), AST, ALT, and glycated haemoglobin (HbA1c), certified by National Glycohemoglobin Standardization Program (NGSP). Full blood count was also assessed on all participants. In addition, an EDTA blood sample was collected and stored at −20 degrees for DNA extraction and analysis.

### 3.3. Genome-Wide DNA Methylation

Genomic DNA was extracted from peripheral blood using the Wizard® Genomic DNA Purification Kit (Promega, Madison, WI, USA) according to the manufacturer's instructions. Briefly, white blood cells were lysed; thereafter, cellular proteins were removed by salt precipitation, and high molecular weight genomic DNA left in solution was then concentrated and desalted by isopropanol precipitation. At least 2 *μ*g of DNA (concentrations ranging between 70 ng/*μ*L and 130 ng/*μ*L) with A260/A280 and A260/A230 ratios ≥ 1.8 was shipped frozen on dry ice, as instructed by Arraystar Inc. (Rockville, MD, USA). Methylated DNA immunoprecipitation (MeDIP) was performed by Arraystar Inc. (Rockville, MD, USA) according to Down et al. [13] with minor modifications as follows.

### 3.4. Sequencing Library Preparation

For MeDIP, genomic DNA was sonicated to ~200–900 bp with a Bioruptor sonicator (Diagenode, Denville, NJ, USA). Thereafter, 800 ng of sonicated DNA was end-repaired, A-tailed, and ligated to single-end adapters following the standard Illumina genomic DNA protocol. After agarose size selection to remove unligated adapters, the adaptor-ligated DNA was used for immunoprecipitation using a human monoclonal anti-5-methylcytosine antibody (Diagenode). For this, DNA was heat-denatured at 94°C for 10 min, rapidly cooled on ice, and immunoprecipitated with 1 *μ*L of primary antibody overnight at 4°C with rocking agitation in 400 *μ*L of immunoprecipitation buffer (0.5% BSA in PBS). To recover the immunoprecipitated DNA fragments, 100 *μ*L of protein G magnetic beads (Life Technologies, Carlsbad, CA, USA) was added and incubated for additional 2 hours at 4°C with agitation. After immunoprecipitation, a total of five immunoprecipitation washes were performed with ice-cold immunoprecipitation buffer. A nonspecific human IgG immunoprecipitation was performed in parallel to methyl DNA immunoprecipitation as a negative control. Washed beads were resuspended in TE buffer with 0.25% SDS and 0.25 mg/mL proteinase K for 2 hours at 65°C and then allowed to cool down to room temperature. MeDIP and supernatant DNA were purified using Qiagen MinElute columns and eluted in 16 *μ*L EB (Qiagen, Germantown, MD, USA). Fourteen cycles of PCR were performed on 5 *μ*L of the immunoprecipitated DNA using the single-end Illumina PCR primers. The resulting reactions were purified with Qiagen MinElute columns, after which a final size selection (300–1,000 bp) was performed by electrophoresis in 2% agarose. Libraries were quality controlled with the Agilent 2100 Bioanalyzer (Agilent Technologies, Santa Clara, CA, USA). An aliquot of each library was diluted in EB (Qiagen) to 5 ng/*μ*L and 1 *μ*L was used in real-time PCR reactions to confirm the enrichment for methylated region. The enrichment of DNA immunoprecipitation was analyzed by qPCR using specific methylated sites at H19 locus and nonmethylated sites at GAPDH.

### 3.5. Sequencing

The library was denatured with 0.1 M NaOH to generate single-stranded DNA molecules and loaded onto channels of the flow cell at 8 pM concentration, amplified* in situ *using TruSeq Rapid SR Cluster Kit (Illumina, San Diego, CA, USA). Sequencing was carried out by running 100 cycles on Illumina HiSeq 2000 according to the manufacturer's instructions. The Agilent 2100 Bioanalyzer was used for accurate assessment of the quality and concentration of the sequencing library, while the size and concentration of each sample were determined after sequencing library preparation.

### 3.6. Data Analysis

After the sequencing platform generated the sequencing images, the stages of image analysis and base calling were performed using Off-Line Basecaller software (OLB V1.8). After passing Solexa CHASTITY quality filter, the clean reads were aligned to the human genome (UCSC HG19) using BOWTIE software (V2.1.0). MeDIP peaks were identified by MACS2 and MAnorm identified DMRs. Statistically significant MeDIP-enriched regions (peaks) detected by MACS2 were identified by comparison to a Poisson background model, using a* q*-value threshold of 10^−2^. The peaks in samples were annotated by the nearest gene (the nearest TSS to the canter of peak region) using the newest UCSC RefSeq database. Peaks were divided into 3 classes on the basis of their distances to UCSC RefSeq genes:

1. *Promoter peaks*: promoters were defined as 2000 bp upstream and downstream from the transcription start site (TSS). Peaks whose centers were located in these promoter regions were defined as promoter peaks.
2. *Gene body peaks*: the gene body region was defined as +2000 bp downstream of the transcription start site (TSS) to the transcription termination site (TTS).
3. *Intergenic peaks*: intergenic regions were defined as the other genomic regions not included in the above 2 regions. Peaks whose centers were located in these intergenic regions were defined as intergenic peaks. MAnorm was used to calculate differentially methylated regions with statistical significance.

### 3.7. Solexa CHASTITY Quality Filter

Individual bases generated from original image files have quality scores, which reflect the probability whether base calling is correct or not. The score is calculated by CHASTITY Formula. The CHASTITY (C) of each base in the short reads is determined by the intensity of four colours (*I*_*A*_,* I*_*C*_,* I*_*G*_, and* I*_*T*_ here), and the formula means “the ratio of the highest (*I*_*C*_ here) of the four (base type) intensities to the sum of highest two (*I*_*C*_ and* I*_*G*_ here).” The CHASTITY (C) should be no less than 0.6 in the first 25 bases.

### 3.8. Gene Ontology (GO) Analysis

The Gene Ontology project provides a controlled vocabulary to describe gene and gene product attributes in any organism (http://www.geneontology.org/). The ontology covers three domains: biological process, cellular component, and molecular function. Fisher's exact test was used to find if there was more overlap between the DE list and the GO annotation list than would be expected by chance. The *P* value denotes the significance of GO terms enrichment in the DE genes. The lower the *P* value, the more significant the GO Term; a *P* value ≤ 0.05 was considered significant.

### 3.9. Pathway Analysis

Pathway analysis is a functional analysis mapping of genes to KEGG pathways. The *P* value (EASE score, Fisher's* P* value, or hypergeometric* P* value) denotes the significance of the pathway correlated to the conditions. The lower the *P* value is, the more significant the pathway is; a *P* value ≤ 0.05 was considered significant.

## 4. Results

### 4.1. General Characteristics of Participants

The general characteristics of the nine female participants are presented in Table 1 for each participant and further summarized across subgroups defined by the glucose tolerance status. All participants had reported no menstrual periods for 6 months or more prior to taking part in this study. As expected from the study design, age and BMI were mostly similar across subgroups, with all participants being obese. Hip circumferences were mostly similar across subgroups, while waist circumference and waist-to-hip ratio decreased with improved glucose tolerance. Blood pressure levels were lowest in normotolerant subjects and highest in those with prediabetes. The lipid profile and indicators of glycaemia improved with improving glucose tolerance status, while fasting insulin levels decreased accordingly (Table 1).

### 4.2. Differentially Methylated Regions

**Figure 1:**
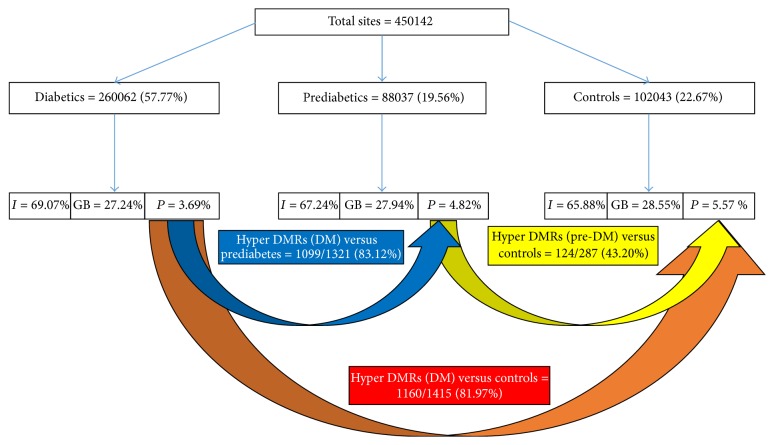
MeDIP-enriched regions (peaks) identified in all the samples. Total sites are the sum of the peak numbers for subjects with diabetes, subjects with prediabetes, and controls and include all the peaks that may be common/redundant across the conditions, as well as the peaks unique to each condition. Distributions of peaks in intergenic (I), gene body (GB), and promoter (P) regions are shown. Statistically significant MeDIP-enriched regions (peaks) were detected by MACS2 and identified by comparison to a Poisson background model, using a* q*-value threshold of 10^−2^. MAnorm was used to calculate differentially methylated regions with statistical significance. The differentially methylated regions located within gene promoters [TSS − 2000 bp; TSS + 2000 bp] are selected and provided. By peak number, there are more hypermethylated peaks in individuals with diabetes (82%) than in the control subjects.

**Figure 2:**
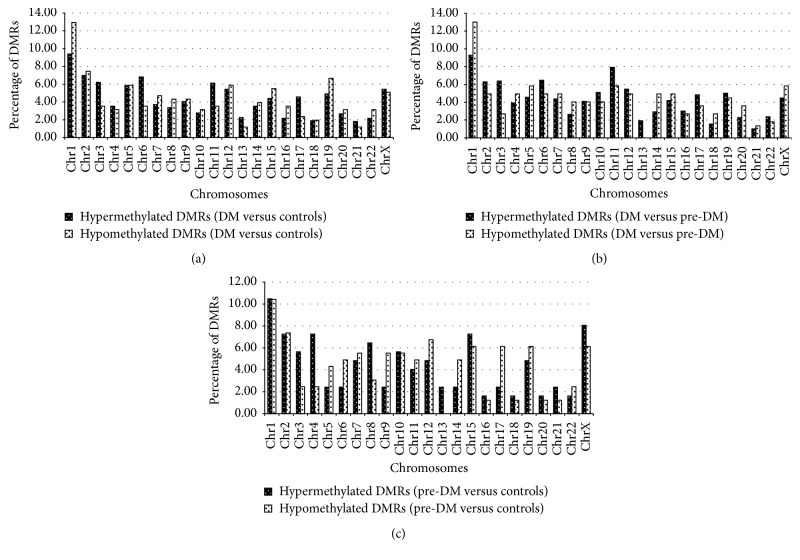
Accumulation of differentially methylated regions (DMRs) in chromosomes. (a) represents DMRs in diabetes versus controls, (b) represents DMRs in diabetes versus prediabetes, and (c) represents DMRs in prediabetes versus controls.

A total of 450,142 statistically significant MeDIP-enriched regions (peaks) were identified in all the samples. As expected, the promoter region [TSS − 2000 bp; TSS + 2000 bp] showed the least number of peaks, compared to the gene body and intergenic regions, in all groups. MAnorm was then used to calculate the statistical significance of differentially methylated regions (DMRs) within gene promoters. Subjects with diabetes showed the highest number of DMRs and these are summarized in Figure 1. Generally, more than 80% of DMRs in subjects with diabetes were hypermethylated when compared to those with prediabetes or normoglycaemia, while no differences were observed between subjects with prediabetes or normoglycaemia (Figure 1). Supplementary Tables 1 to 6 in Supplementary Material available online at http://dx.doi.org/10.1155/2016/3172093 show the DMRs in subjects with diabetes, subjects with prediabetes, and controls. To summarize these data, we grouped the DMRs according to chromosomal location and this is shown in Figure 2. Compared to controls and subjects with prediabetes, hypermethylated DMRs in subjects with diabetes were more common in chromosomes, 3, 6, 11, 13, and 17, while in chromosome one, there were more hypomethylated DMRs (Figure 2). No hypomethylated DMRs were present in chromosome 13 in subjects with diabetes or prediabetes when compared with each other or when those with prediabetes were compared to controls (Figure 2).

### 4.3. Pathway Analysis

**Figure 3:**
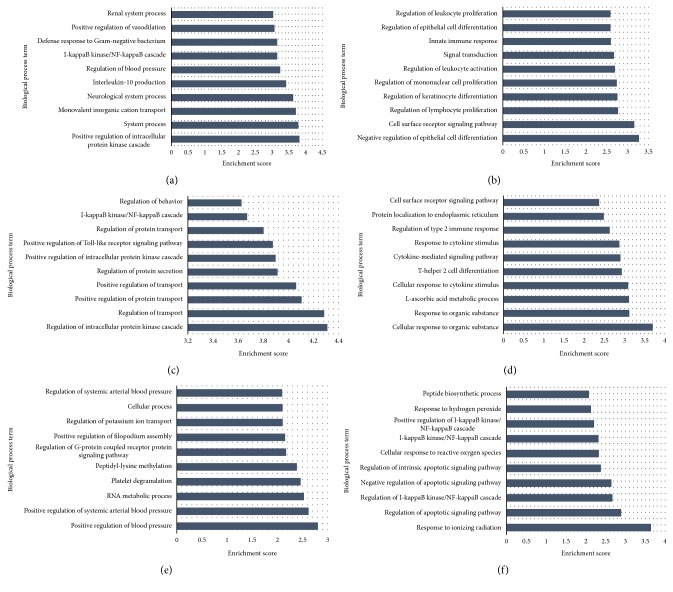
GO analysis of the differentially methylated genes in diabetes, prediabetes, and controls. The bars plot shows the top ten enrichment score values of the significant enrichment terms.* X*-axis: GOID's enrichment score value; it equals −log 10(*P* value);* Y*-axis: GO category. (a) Hypomethylated diabetes versus controls. (b) Hypermethylated diabetes versus controls. (c) Hypomethylated diabetes versus prediabetes. (d) Hypermethylated diabetes versus prediabetes. (e) Hypomethylated prediabetes versus controls. (f) Hypermethylated prediabetes versus controls.

We first performed Gene Ontology (GO) classification to retrieve the biological process, cellular process, and molecular function of the DMRs and these are presented in Supplementary Tables 2–24, while Figure 3 shows biological processes in the top 10 enrichment scores for DMRs in subjects with diabetes or prediabetes. As shown in Figure 3, these hypermethylated DMRs in subjects with diabetes or prediabetes were widely associated with cell surface receptor signaling and inflammatory pathways. In addition, glucose transport, WNT signaling, muscle development, pancreas development genes, and insulin signaling pathway were associated with hypermethylation in subjects with diabetes or prediabetes (Supplementary Tables 7–12). Although the I-kappaB kinase/NF-kappaB cascade was associated with hypomethylated DMRs in subjects with diabetes and hypermethylated DMRs in subjects with prediabetes, the genes associated with these pathways were different in each group. For example, in subjects with prediabetes, the hypermethylated genes were CHUK, TRIM38, PLK2, TNFRSF19, and ZMYND11, while in subjects with diabetes they were BCL3, IL23A, F2RL1, S100A12, TNFRSF10B, NEK6, RNF31, SLC35B2, and IRAK1BP1. Pathway analysis based on the functional analysis mapping of genes to KEGG pathways also showed an association with inflammatory pathways (Table 2). The linoleic acid metabolism and arachidonic acid metabolism pathways were progressively hypomethylated from prediabetes to diabetes. On the other hand, the hypertrophic cardiomyopathy (HCM) pathway was associated with hypermethylated DMRs in subjects with diabetes when compared to either controls or subjects with prediabetes.

## 5. Discussion

Emerging data supports the role of epigenetic mechanisms in the development of diabetes; however, to date, genome-wide DNA methylation profiling has not involved subjects with prediabetes or diabetes from sub-Saharan Africa or Africa in general. In this preliminary genome-wide DNA methylation analysis of individuals with prediabetes or diabetes from South Africa, we provide DMRs data and their biological pathways that appear to be affected in subjects with diabetes or prediabetes, as well as those that appear to show a trend from the prediabetes state to diabetes. For instance, the linoleic acid metabolism and arachidonic acid metabolism pathways were associated with hypomethylated DMRs in subjects with prediabetes versus controls and in those with diabetes versus those with prediabetes, suggesting that the hypomethylation of these genes is likely to be an early process that occurs before the onset of overt diabetes. Our data also shows that genes related to the immune system, signal transduction, glucose transport, and pancreas development are hypermethylated in subjects with prediabetes or diabetes. When they are investigated further, these DMRs may be potential biomarkers of disease occurrence/progression and also could suggest possible targets for the development of new treatments.

Whole epigenetic profiling in individuals with type 2 diabetes is relatively in its infancy with the first study reported in 2012. The study was conducted using pancreatic islets of 5 individuals with type 2 diabetes and identified 276 DMRs, where 96% of the 254 DMRs located in the promoter region were hypomethylated [14]. The DMRs were associated with beta-cell function, cell death, and adaptation to metabolic stress. Similar DMRs (71) to Volkmar et al. [14] have recently been reported in a study, where they were associated with pathways in cancer, axon guidance (SEMA4A and SEMA5B), MAPK signaling (CACNA1H) focal adhesion (ITGB4), ECM-receptor interaction (AGRN and TGB4), and actin cytoskeleton (TGB4) [7]. Furthermore, the study showed an increased accumulation of the DMRs in chromosomes 1 and 2, while in chromosome 19, DMRs were lessened [7]. In the current study, we found a total of 1415 DMRs in the promoter regions of subjects with diabetes when compared to control subjects and 81.7% of these were hypermethylated. Similar to Dayeh et al.'s [7] report, the DMRs were mostly accumulated in chromosomes 1 and 2 but were least in chromosome 21 in subjects with diabetes compared to controls in our study. We also observed similar finding between subjects with prediabetes and controls showing higher accumulation in chromosomes 1 and 2, while chromosomes 13, 16, 18, 20, 21, and 22 had less than 10 DMRs. A longitudinal study that investigated hypomethylated DMRs showed that progression from normoglycaemia to a worse glucose tolerance state was associated with early differential methylation prior to disease manifestation [15]. The authors analyzed methylation levels of candidate DMRs identified by whole epigenetic profiling in 62 subjects with impaired glucose metabolism and 64 controls who maintained a normal glucose tolerance status during follow-up and demonstrated significantly lower percent of methylation before the appearance of the disease in those that progressed [15]. Similarly, we have also observed common DMRs as well as common pathways in prediabetes and diabetes individuals' inflammatory genes including the lipid metabolism pathway, which appeared to be progressively modified from prediabetes state to diabetes. We observed that the arachidonic acid (AA) pathway was hypomethylated and appeared to be modified in prediabetic and diabetic states; however, different isoforms of the genes were involved in each glycaemic state. For example, in subjects with prediabetes versus controls, cytochrome P450, family 4, subfamily F, polypeptide 3 (CYP4F3), CYP4F8, Phospholipase A2, group IIC (PLA2G2C), and PLA2G4E were differentially methylated, while in subjects with diabetes versus prediabetes, CYP2E1 and PLA2G12A were involved.

Lipids are important components of all mammalian cells and have a variety of biological functions, including serving as energy reservoirs and mediators of inflammation known as oxylipins. Oxylipins result from the oxygenation of PUFAs by three types of enzymes, cyclooxygenases, lipoxygenases, and cytochrome P [16]. The type of PUFA oxidized and enzyme involved determine the production of oxylipins. The arachidonic acid (AA) generates most of the inflammatory molecules involved in cell signaling cascades and is a precursor of eicosanoids. Eicosanoids include prostaglandins (PGAs), leukotrienes (LTs), and thromboxanes (TXAs) and their role is dependent on the type of fatty acid from which they are derived [17]. In this regard, we also observed differentially methylated signals of cytochrome P450 and Phospholipase A2 (PLA2). Inflammatory activation of the PLA2 enzyme promotes the release of AA from cell membranes phospholipids, which in turn is metabolized by cyclooxygenases (COX), yielding eicosanoids [17]. Taken together, the epigenetic modifications of oxylipins indirectly represent a chronic inflammatory pathway involvement in diabetes development. Because fatty acids are derived from the diet in the form of linoleic acid, followed by desaturation and elongation into specific fatty acids, the elucidation of lipid pathway epigenetics may contribute to the formulation of treatment and prevention strategies. For instance, supplement studies using AA, EPA, and/or ALA have demonstrated an effect on the production of eicosanoids and inflammatory markers. In a study comprising healthy males, 1.5 g of AA increased PGE2 and leukotriene LTB4 [18], while supplementation with fish oil containing EPA and DHA decreased the generation of TNF*α* and IL-1 by 70% and 78%, respectively [19]. Moreover, epigenetic changes are reversible to an extent that in some neurological diseases and cancers epigenetic drugs have been proposed or are currently being used [20, 21]. In cancer, for instance, methylation inhibiting drugs include cytidine analogs such as 5-azacitidine [22] and zebularine [23]. In view of the above, it is clear that epigenetics do offer tremendous opportunities for treatment and management of diseases.

Considering the current published literature on the epigenetics of type 2 diabetes globally, it is encouraging to note that a few whole epigenomic studies have been conducted in populations from Africa. These include studies that investigated the effect of environment in Moroccans, Ethiopians, and Egyptians [24–26], severe bladder damage in Ghanaians [27], and exposure to famine in offspring from Gambia [28]. Epigenetic determinants have been shown to differ between populations. For example, in HapMap lymphoblastoid cell lines derived from individuals of European or African ancestry, population-specific cytosine modifications in samples derived from Yoruba people from Ibadan, Nigeria, and Caucasian residents of European ancestry from Utah were observed [29]. The differences between and within population groups have been linked to population-disease-specific single nucleotide polymorphisms (SNPs). In diabetes, for instance, 17 of the 40 type 2 diabetes candidate genes identified by genome-wide association studies (GWAS) were differentially methylated in pancreatic islets of subjects with diabetes [7]. Interestingly, in our study, KEGG pathway analysis identified hypomethylated DMRs in subjects with diabetes that were associated with African trypanosomiasis pathway. African trypanosomiasis is a sleeping sickness caused by* Trypanosoma* species from Africa, suggesting a population-specific selection of DNA methylation in this population with an African ancestry.

In this study, DNA methylation was investigated using whole genome MeDIP sequencing (MeDIP-Seq); though bisulfite sequencing is currently considered the gold standard for detecting DNA methylation, it does not distinguish between 5-methylcytosine (5mC) and 5-hydroxymethylcytosine (5hmC). While MeDIP-Seq obviates the need for bisulfite treatment of DNA, a limitation of the technique is the inability to detect individual differentially methylated CpG sites. A major limitation of this study is the lack of verification of regions using other sequencing-based DNA methylation profiling methods. However, a quantitative comparison of four sequencing-based DNA methylation methods including MeDIP-Seq demonstrated comparable methylation calls of all four methods but differences in CpG coverage, resolution, quantitative accuracy, efficiency, and cost [30]. Other limitations of the study include the one gender and small number of individuals investigated. The pancreatic *β*-cells are believed to be the ideal tissue for type 2 diabetes epigenetics. We made use of peripheral white blood cells and it has been shown that DNA methylation is different between blood cell types [31]; thus our findings should be interpreted with caution. While we carefully matched the participants, we take note that the smoking patterns were not similar between the groups. Therefore, it is likely that some of the DMRs observed are not necessarily due to diabetes or prediabetes but perhaps nicotine. Similarly, all participants were obese (BM1 > 30 kg/m^2^); we cannot dismiss the fact that some DMRs are obesity related. Although the population investigated is from Africa, it is noteworthy to mention that this is a unique heterogeneous group, which is of mixed genetic origin with contributions from Europeans, South Asians, Indonesians, and a population genetically close to the isiXhosa sub-Saharan Bantu population [32]. Therefore we cannot rule out the possible role of genetic ancestral components in the methylation patterns observed. However, financial constraints and the technical complexity of generating ancestry informative markers, particularly for this South African population group, still remain a challenge.

In conclusion, our study provides basis for candidate methylation analysis in Africa. Considering that GWAS studies of diabetes involving populations from Africa are not available, we recommend methylation quantitative trail loci (meQTL) investigations coupled with ancestry informative markers to account for population stratification.

## Supplementary Material

Supplementary Tables 1 to 24 is an excel file. S1 to S6 shows DMRs within gene promoters in prediabetes vs controls (S1 and S2); diabetes vs controls (S3 and S4); diabetes vs prediabetes (S5 and S6). BP1 to BP6 depicts Gene Ontology (GO) classification of biological processes in diabetes vs controls (BP1 and BP2); diabetes vs prediabetes (BP3 and BP4); prediabetes vs controls (BP5 and BP6). CP1 to CP6 shows GO classification of cellular process in diabetes vs controls (CP1 and CP2); diabetes vs prediabetes (CP3 and CP4); prediabetes vs controls (CP5 and CP6). MF1 to MF6 shows GO classification of molecular function in diabetes vs controls (MF1 and MF2); diabetes vs prediabetes (MF3 and MF4); prediabetes vs controls (MF5 and MF6).

## Figures and Tables

**Table 1:** Characteristics of all participants.

	Diabetes				Prediabetes				Controls			
	Case 1	Case 2	Case 3	Mean	Case 1	Case 2	Case 3	Mean	Case 1	Case 2	Case 3	Mean
Age, years	62	52	52	55.3	52	50	55	52.3	62	52	52	55.3
BMI, kg/m^2^	35.3	39.3	31.2	35.3	32.6	35.2	30.4	32.7	41.3	34.0	31.7	35.6
WaistC, cm	120	103	116	113	104	108	116	109	112	93	89	98
HipC, cm	118	124	113	118	108	119	129	118	127	124	106	119
WHR	1.02	0.83	1.02	0.96	0.96	0.91	0.90	0.93	0.88	0.75	0.84	0.82
SBP, mmHg	153	183	143	160	178	164	163	168	142	127	137	135
DBP, mmHg	78	130	61	90	109	101	78	96	72	75	92	80
S-Creat., *μ*mol/L	45	75	35	52	69	47	52	56	62	58	50	57
GGT, U/L^*∗*^	18	42	295	118	22	20	12	18	22	20	99	47
ALT, U/L	19	86	61	55	22	16	17	18	12	12	30	18
AST, U/L	24	45	50	40	21	18	18	19	21	16	30	22
Smoking, yes/no	Yes	Yes	Yes		Yes	No	No		Yes	Yes	No	
Cotinine, ng/mL^*∗*^	160	>500	76.3	245.4	94	<10	<10	38	94.4	338	<10	147.5
TC, mmol/L	6.0	6.0	8.3	6.8	4.9	4.1	5.5	4.8	3.6	5.2	4.7	4.5
TG, mmol/L	1.49	3.35	2.51	2.45	2.10	1.21	1.04	1.45	1.22	1.23	1.27	1.24
HDL-C, mmol/L	1.09	1.1	1.87	1.35	1.09	1.17	1.42	1.23	1.35	1.08	1.42	1.28
LDL-C, mmol/L	3.9	4.2	5.1	4.4	3.1	2.1	3.5	2.9	1.6	3.4	2.7	2.6
FSI, mIU/L	97.9	68.7	47.7	71.4	77.3	29.9	110.2	72.5	72.8	20.8	30.1	41.2
FSI, mIU/L^*∗*^	20.7	21.2		21.0	5.7	5.5	11.5	7.6	10.4	7.6	5.3	7.8
HbA1c, %	7.2	7.5	6.1	6.9	5.0	6.1	6.4	5.8	5.9	5.6	6.1	5.9
HbA1c, mmol/mol	55.2	58.5	43.2	52.3	31.1	43.2	46.4	40.2	41.0	37.7	43.2	40.6
FBG, mmol/L	9.0	8.1	7.8	8.3	4.7	6.2	6.1	5.7	5.4	2.8	5.8	4.7
2-hour PG, mmol/L	16.4	13.8	5.9	12.0	7.8	10.3	9.4	9.2	7.4	5.0	6.1	6.2
U-CRP, mg/L	3.2	10.4	5.3	6.3	5.2	15.6	21.6	14.1	23.4	8.2	5.9	12.5
RBC, ×10^12^/L	4.56	5.07	4.37	4.67	4.86	4.42	4.88	4.72	3.75	4.42	4.82	4.33
Platelet count, ×10^9^/L	284	217	369	290	238	286	305	276	281	152	256	230
WCC, ×10^9^/L	8.6	10.0	7.8	8.8	7.1	8.0	8.9	8.0	6.0	8.3	4.6	6.3
Hb, g/dL	13.0	15.1	12.6	13.6	13.6	13.2	13.5	13.4	6.1	14.4	13.6	11.4
Haematocrit, L/L	0.39	0.46	0.37	0.41	0.42	0.39	0.41	0.41	0.22	0.42	0.41	0.35
MCV, fL	85	90	85	87	86	89	83	86	57	95	86	79
MCH, pg	29	30	29	29	28	30	28	29	16	33	28	26
MCHC, g/dL	34	33	34	34	33	34	33	33	29	34	33	32
RDW, %	14.0	15.0	15.2	14.7	12.7	14.1	14.0	13.6	25.3	14.8	15.9	18.7
Neutrophils, ×10^9^/L	5.6	7.2	4.6	5.8	4.5	4.5	5.4	4.8	2.8	5.7	2.9	3.8
Lymphocytes, ×10^9^/L	2.3	2.0	2.4	2.2	2.1	3.0	2.8	2.6	2.3	1.9	1.4	1.9
Monocytes, ×10^9^/L	0.50	0.30	0.40	0.40	0.30	0.30	0.30	0.30	0.72	0.46	0.20	0.46
Eosinophils, ×10^9^/L	0.10	0.40	0.40	0.30	0.20	0.20	0.40	0.27	0.12	0.14	0.10	0.12
Basophils, ×10^9^/L	0.01	0.01	0.10	0.04	0.01	0.01	0.01	0.01	0.06	0.04	0.01	0.04

2-hour PG, after 2-hour plasma glucose; 2-hour SI, after 2-hour serum insulin; ALT, alanine aminotransferase; AST, aspartate aminotransferase; DBP, diastolic blood pressure; FBG, fasting plasma glucose; FSI, fasting serum insulin; GGT, gamma glutamyl transpeptidase; Hb, haemoglobin; HDL-C, high density lipoprotein cholesterol; HipC, hip circumference; LDL-C, low density lipoprotein cholesterol; MCH, mean cell haemoglobin; MCHC, mean corpuscular haemoglobin concentration; MCV, mean cell volume; RBC, red blood cell count; RDW, red cell distribution width; S-Creat., serum creatinine; SBP, systolic blood pressure; TC, total cholesterol; TG, triglycerides; U-CRP, ultrasensitive C-reactive protein; WaistC, waist circumference; WCC, white cell count; WHR, waist-to-hip ratio.

*∗* refers to using the median instead of mean because, since the data is skewed for those parameters, the mean will give a wrong meaning.

**Table 2:** KEGG analysis of top ten enrichment score (−log⁡10(*P* value)) values of the significant enrichment pathway.

Pathway ID	Definition	Fisher's *P* value	Enrichment score	Genes
*Hypomethylated diabetes *versus* control*				
hsa05143	African trypanosomiasis	0.0073	2.133882	F2RL1//HPR//THOP1
hsa05340	Primary immunodeficiency	0.0086	2.064522	AIRE//CD8A//IGLL1
hsa04640	Hematopoietic cell lineage	0.0200	1.697467	CD1B//CD5//CD8A//CD9
hsa04970	Salivary secretion, *Homo sapiens*	0.0216	1.665411	ADRB2//CST5//KCNN4//NOS1
hsa00670	One carbon pool by folate	0.0229	1.639815	ATIC//MTFMT
hsa04977	Vitamin digestion and absorption	0.0322	1.490856	SLC19A2//SLC23A1

*Hypermethylated diabetes *versus* control*				
hsa00910	Nitrogen metabolism	0.0015	2.818773	CA13//CA5B//CA9//CPS1//GLU
				D2
				APLN//CHRNA1//CHRNA5//CH
				RNB3//CNR1//EDN1//EDNRB//
				FSHB//GABRA6//GABRG2//GA
				BRR1//GCG//GHR//GPR156//G
				RIA1//GRM4//HCRTR2//LGR4//
				NMUR2//NPFF//NPY1R//P2RX6
hsa04080	Neuroactive ligand-receptor interaction	0.0207	1.683002	//P2RY14//PLG//RXFP1//TAC4
hsa00524	Butirosin and neomycin biosynthesis	0.0254	1.594577	GCK//HK2
	Glycosaminoglycan biosynthesis, heparan			EXT1//EXTL2//HS3ST5//XYLT
hsa00534	Sulfate/heparin	0.0361	1.441751	1
				CACNB2//EDN1//IGF1//ITGA1//
				ITGB1//MYH6//PRKAG2//PRK
hsa05410	Hypertrophic cardiomyopathy (HCM)	0.0362	1.441099	AG3//TPM1
hsa00750	Vitamin B6 metabolism	0.0368	1.433896	PDXP//PSAT1
hsa04950	Maturity onset diabetes of the young	0.0413	1.384024	GCK//HNF1A//NR5A2//PDX1

*Hypomethylated diabetes *versus* prediabetes*				
hsa00770	Pantothenate and CoA biosynthesis	0.0137	1.86211	COASY//PANK2
hsa04640	Hematopoietic cell lineage	0.0141	1.850323	CD14//CD1A//CD1B//CD3E
hsa00590	Arachidonic acid metabolism	0.0304	1.516647	CYP2E1//GGT1//PLA2G12A
hsa03320	PPAR signaling pathway	0.0369	1.433374	ACSL4//NR1H3//PCK2
hsa00591	Linoleic acid metabolism	0.0378	1.42268	CYP2E1//PLA2G12A

*Hypermethylated diabetes *versus* prediabetes*				
hsa05410	Hypertrophic cardiomyopathy (HCM)	0.0063	2.201901	CACNA1S//DMD//ITGA4//MY
				H6//PRKAB2//PRKAG3//TGFB3
				//TNF//TNNI3//TPM4
				ACVR1B//CCR6//CCR8//CX3C
				R1//CXCL1//CXCL9//EDAR//G
				HR//IFNA2//IFNA21//IL18RAP//
				IL3//IL3RA//IL4R//IL9//TGFB3//
				TNF//TNFRSF10D//TNFRSF14//
hsa04060	Cytokine-cytokine receptor interaction	0.0085	2.071022	TNFRSF9//TNFSF18//TNFSF8
hsa03060	Protein export	0.0210	1.678045	SEC61G//SRP9//SRPR//SRPRB
				ADCY4//CACNA1S//DMD//ITG
				A4//MYH6//TGFB3//TNF//TNNI
hsa05414	Dilated cardiomyopathy	0.0251	1.600573	3//TPM4
				ADCY4//ANAPC11//ATF1//ATF
				3//CCNB2//CREM//HLA-
				A//HLA-
				E//IKBKB//KRAS//MAD2L1//M
				RAS//MSX2//NFYB//TGFB3//T
hsa05166	HTLV-I infection	0.0382	1.418455	LN2//TNF//WNT16//WNT5B
				ACSL6//IKBKB//PRKAB2//PRK
hsa04920	Adipocytokine signaling pathway	0.0455	1.342336	AG3//PTPN11//SOCS3//TNF

*Hypomethylated prediabetes *versus* control*				
hsa00590	Arachidonic acid metabolism	0.0026	2.593265	CYP4F3//CYP4F8//PLA2G2C//P
				LA2G4E
hsa00592	Alpha-linolenic acid metabolism	0.0211	1.676649	PLA2G2C//PLA2G4E
hsa00591	Linoleic acid metabolism	0.0278	1.555232	PLA2G2C//PLA2G4E
				MYLK3//PLA2G2C//PLA2G4E//
hsa04270	Vascular smooth muscle contraction	0.0301	1.521206	RAMP3
hsa04350	TGF-beta signaling pathway	0.0352	1.453226	LEFTY2//SP1//TGFB3

*Hypermethylated prediabetes *versus* control*				
hsa05202	Transcriptional misregulation in cancer	0.0190	1.720526	CCNT1//ELK4//EYA1//RARA
hsa05221	Acute myeloid leukemia	0.0425	1.371986	CHUK//RARA
